# IFE-CMT: Instance-Aware Fine-Grained Feature Enhancement Cross Modal Transformer for 3D Object Detection

**DOI:** 10.3390/s25185685

**Published:** 2025-09-12

**Authors:** Xiaona Song, Haozhe Zhang, Haichao Liu, Xinxin Wang, Lijun Wang

**Affiliations:** School of Mechanical Engineering, North China University of Water Resources and Electric Power, Zhengzhou 450045, China; songxiaona1@126.com (X.S.); 201911116@stu.ncwu.edu.cn (H.Z.); liuhaichao@ncwu.edu.cn (H.L.); wangxinxin@ncwu.edu.cn (X.W.)

**Keywords:** 3D object detection, multi-modal fusion, LIDAR point cloud, multi-view images, autonomous driving

## Abstract

In recent years, multi-modal 3D object detection algorithms have experienced significant development. However, current algorithms primarily focus on designing overall fusion strategies for multi-modal features, neglecting finer-grained representations, which leads to a decline in the detection accuracy of small objects. To address this issue, this paper proposes the Instance-aware Fine-grained feature Enhancement Cross Modal Transformer (IFE-CMT) model. We designed an Instance feature Enhancement Module (IE-Module), which can accurately extract object features from multi-modal data and use them to enhance overall features while avoiding view transformations and maintaining low computational overhead. Additionally, we design a new point cloud branch network that effectively expands the network’s receptive field, enhancing the model’s semantic expression capabilities while preserving texture details of the objects. Experimental results on the nuScenes dataset demonstrate that compared to the CMT model, our proposed IFE-CMT model improves mAP and NDS by 2.1% and 0.8% on the validation set, respectively. On the test set, it improves mAP and NDS by 1.9% and a 0.7%. Notably, for small object categories such as bicycles and motorcycles, the mAP improved by 6.6% and 3.7%, respectively, significantly enhancing the detection accuracy of small objects.

## 1. Introduction

Three-dimensional object detection tasks aim to accurately identify and classify objects in three-dimensional scenes, playing a critical role in the safety of intelligent vehicles [1,2,3]. Current approaches to 3D object detection in autonomous driving scenarios can be broadly categorized into vision-based methods, LIDAR point cloud-based methods, and vision-point cloud fusion methods [4]. Table 1 summarizes the advantages, disadvantages, and corresponding references of these methods for reference.

Although visual methods can obtain rich scene semantic information, pure vision-based 3D detection methods perform poorly due to the lack of accurate spatial information. LIDAR point cloud data can directly reflect the position and scale information of each object in a large-scale scene [17], thereby helping the model to accurately locate objects. However, due to the sparsity and non-semantic nature of point cloud data [18], improvements in the accuracy of point cloud-based 3D detection tasks have reached a bottleneck. Multi-modal 3D detection that fuses visual and point cloud information capitalizes on the complementary characteristics of both modalities. This approach utilizes semantic information derived from images to enhance representations obtained from point clouds while leveraging spatial details from point clouds to address the deficiencies related to depth perception found in images.

Currently, the majority of multi-modal fusion algorithms convert point cloud features and image features into a unified Bird’s Eye View (BEV) representation [19]. This approach facilitates feature fusion and detection tasks, as exemplified by models such as AVOD-Net [20], BEV-Fusion [21,22], and EA-LSS [23]. This method is affected by multi-sensor calibration and explicit view alignment errors, which may lead to a decrease in accuracy. SparseFusion [24] avoids dense BEV feature transformations and instead uses a sparse view transformer to generate sparse BEV features for image regions of higher confidence, achieving excellent detection accuracy and demonstrating the critical role of sparse instance features in detection results. The Cross Model Transformer [15] (CMT) implicitly achieves multi-modal feature alignment through coordinate encoding, eliminating the need for explicit view transformations, reducing memory usage, and avoiding the biases introduced by view transformations. It also exhibits stronger robustness to sensor configurations. Therefore, this paper selects the CMT method as the baseline model.

Existing multi-modal models have achieved high detection accuracy, especially for noticeable objects such as cars. However, most of these methods only focus on fusing overall scene information. During complex view transformations and feature fusion processes, fine-grained features are significantly lost, making it difficult for the model to learn more subtle feature differences between different categories. This leads to reduced detection accuracy for small objects and objects with similar features, such as bicycles and motorcycles. Therefore, enhancing the model’s ability to capture fine-grained information and improving the detection accuracy of various object categories are crucial for enhancing the model’s reliability in practical applications.

In this paper, we proposed a multi-modal 3D object detection model named IFE-CMT that enhances the model’s ability to represent sparse object in scene features. By implementing an instance-aware fine-grained feature enhancement strategy, the model’s detection performance for small and easily confused objects is improved. First, we designed an Instance feature Enhancement module (IE-Module), which utilizes instance heatmaps to extract object features from multi-modal features and applies them to enhance point cloud scene features through an attention mechanism, providing the model with a more refined feature representation. Second, based on the characteristic that 3D point cloud data has no significant scale transformations [25,26], we designed a novel neck network that combines the Feature Pyramid Network [27] (FPN) and Atrous Spatial Pyramid Pooling [28] (ASPP) architectures, enabling the model to expand its receptive field while reserving multi-scale features. Finally, we additionally introduced Spatial and Channel reconstruction Convolution [29] (SCConv) to reduce redundant features in the network and improve computational efficiency.

The contributions of this paper are as follows: (1) We designed a lightweight and easy-to-deploy Instance feature Enhancement module (IE-Module), which does not require explicit feature alignment. This module can efficiently couple sparse instance features with scene features and is easy to use in any multi-modal/mono-modal model. By introducing an instance heatmap loss function, it can be plug-and-play, significantly improving model performance while adding only 0.2s to inference time. (2) We improved the point cloud branch network architecture to make it more suitable for extracting point cloud features. By combining Feature Pyramid Network (FPN) with Atrous Spatial Pyramid Pooling (ASPP) neck architecture, the network can expand its receptive field while retaining shallow texture detail information, enhancing the network’s semantic and detail representation. In addition, we introduced Spatial and Channel reconstruction Convolution (SCConv) to reduce feature redundancy in spatial and channel dimensions, improving the model’s learning efficiency. (3) Experimental results on the nuScenes dataset show that, compared to CMT, our IFE-CMT improves mAP and NDS by 2.1% and 0.8% on the validation set, respectively. And, it also improves mAP and NDS by 1.9% and 0.7% on the test set. Notably, detection performance is significantly enhanced for categories such as bicycles and motorcycles.

## 2. Related Work

### 2.1. Visual 3D Object Detection

Current pure vision-based 3D object detection research primarily focuses on multi-view stereo images methods [30], which can be simply categorized into BEV methods and non-BEV methods. BEV methods convert image features to Bird’s Eye View (BEV) space through spatial range transformations; a BEV perspective is more conducive to predict object location. LSS [5] predicts depth distributions and performs per-pixel outer products with image features, thereby converting image features into frustum features, which are then transformed into the BEV perspective using camera intrinsics. BEVDet [31] extracts image features and performs view transformations via LS; it specially designs an efficient BEV-Encoder to accurately perceive key information such as size and direction. However, the accuracy of depth estimation remains unsatisfactory. BEVDepth [6] adds a separate depth correction subnetwork, utilizing real LIDAR depth information to enhance the model’s depth estimation capabilities during training. BEV-Former [32] obtains implicit depth information through a cross-attention mechanism and adds temporal data to effectively mitigate the issue of low depth estimation accuracy. BEV methods face challenges such as feature loss during geometric transformations and high memory consumption.

Non-BEV methods do not perform view transformations, thereby avoiding information loss during the process and reducing computational complexity. DETR3D [7] builds upon DETR [33] by introducing an ensemble prediction module, which bridges 2D and 3D computations. This module projects 3D reference point information onto multi-view images via camera intrinsic and extrinsic parameters, enabling separate 3D predictions for each object. PETR [8] further proposes 3D position encoding, allowing reference points to be updated directly in the 3D semantic space, thereby eliminating the need for multi-view projection. CMT also utilizes reference points in 3D space for position encoding and query generation. PETR-V2 [34] further improves model performance by introducing temporal modeling and feature-weighted position encoding. MV2D [35] first uses a 2D detector to obtain 2D detection results, then combines these results with image features and camera parameters to dynamically generate objects’ query information, and decorates object features with 3D position encoding.

Visual 3D object detection achieves high inference speed and low cost, but the lack of spatial information results in poor detection accuracy.

### 2.2. LIDAR 3D Object Detection

Point cloud data in autonomous driving scenarios is typically obtained from LIDAR and can be mathematically described as a set of discrete points in three-dimensional space [1]. Unlike image data, point cloud data does not have a fixed data structure or resolution. It directly reflects the true coordinates and reflectance intensity of each object in the scene, possessing inherent spatial information. However, its irregular nature also makes it challenging to direct process it.

The voxelization approach effectively addresses this issue. VoxelNet [36] divides the point cloud space into regular voxel cells and performs pooling on the points within each voxel to represent the voxel features. By setting different voxel resolutions, it can learn multi-scale features. This approach converts point clouds into regular data inputs without losing point cloud spatial information, but it perform high content occupancy. To address this issue, the SECOND [9] model introduces sparse encoding convolutions, processing only dense voxel features, and proposes an input–output rule index matrix (rulebook) to map dense features back to sparse features. As a result, SECOND achieves a balance between inference speed and accuracy. Additionally, PointPillars [37] divides point clouds into point pillars without distinguishing by height, performs feature extraction on each pillar and generates pseudo-images. This approach achieves highly efficient detection speed while maintaining detection quality. PillarNeXt [38] considers the long-range and scale-free transformation characteristics of point cloud data, using Atrous Spatial Pyramid Pooling (ASPP) to increase the receptive field instead of the commonly used Feature Pyramid Network (FPN) structure, and has achieved performance improvements. These voxelization models have become commonly used point cloud backbone networks in subsequent point cloud or multi-modal 3D detection research.

With the tremendous success of the Transformer [39] architecture in visual detection, its excellent long-range modeling and context information capture capabilities have quickly led to its application in point cloud 3D detection [40]. PointFormer [11] extends the Transformer architecture to point cloud object detection tasks, introducing the Local Transformer, Local–Global Transformer, and Global Transformer to compute local features, multi-scale features, and global features with contextual relationships, respectively. Inspired by the success of the DETR architecture, 3DETR [12] extends it to point cloud 3D detection by randomly sampling reference points in three-dimensional space and applying Fourier position encoding for processing, achieving significant success. However, while Transformer methods have achieved leading detection accuracy, their high computational complexity and low detection speed have somewhat constrained further performance improvements. Current research in point cloud 3D detection primarily focuses on balancing speed and accuracy. VoxelNeXt [10] and SAFDNet [41] have designed detectors based on fully sparse convolutions, while DSVT [42] has introduced a dynamic sparse window attention mechanism. These methods effectively reduce computational overhead.

Point cloud 3D object detection has achieved high detection accuracy, but the sparse and non-semantic nature of point cloud data result in accuracy bottlenecks. Additionally, the currently used network architectures in point cloud 3D object detection often follow successful approaches in the 2D object detection, such as Feature Pyramid Network (FPN). That means the performance of point cloud models has not been fully exploited and can be improved with simple modifications. This paper combines the Atrous Spatial Pyramid Pooling (ASPP) network and FPN network to improve the point cloud branch architecture, effectively expanding the receptive field while preserving the detailed information of shallow-layer features, thereby optimizing the overall feature representation of the network. Finally, Spatial and Channel reconstruction Convolution (SCConv) is introduced to reduce feature redundancy in the backbone network and improve learning efficiency.

### 2.3. Vision-Point Cloud Fusion 3D Object Detection

Vision-point cloud fusion 3D detection algorithms have achieved excellent accuracy performance. The combination of the two modalities compensates for the lack of depth information in image data and the insufficient of semantic information in point cloud data [43]. Depending on the fusion stage, vision-point cloud fusion can be divided into data-level fusion, feature-level fusion, and decision-level fusion.

Data-level fusion often uses images to provide prior information for point clouds. For example, F-PointNets [13] converts 2D proposals into 3D frustums and performs instance segmentation on the point cloud within the frustums to obtain object information. The MVP [14] model generates virtual points by projecting 2D object masks into the LIDAR space to enhance point cloud representation. Data-level fusion directly combines the two modalities, preserving the characteristics of each data type to the greatest extent. However, its fusion method is overly reliant on visual detection results, leading to suboptimal accuracy performance.

Feature-level fusion is a hot topic in multi-modal fusion research. It uses image backbone and point cloud backbone to extract two types of features, and achieves feature fusion through designed feature interaction strategies, and achieves the best performance. MV3D [44] converts point cloud features into Bird’s Eye View (BEV) features and front view (FV) features, then combine them with image RGB features to improve accuracy performance. AVOD-Net [20] removes FV features from this foundation, demonstrating that the combination of BEV features and RGB features is sufficient to accurately represent 3D scene information, laying the groundwork for subsequent feature-level fusion research. On this basis, Transfusion [16] achieves finer-grained feature fusion through a multi-head attention mechanism. Based on LSS technology, BEV-Fusion [21,22] converts image features and point cloud features into a unified BEV feature representation, reducing computational complexity through pre-computation and BEV pooling techniques, achieving state-of-the-art (SOTA) performance. SparseFusion [24] abandons dense BEV feature fusion, using geometric and semantic transformers to complement image and point cloud features, respectively, extracting candidates from point clouds and images for sparse fusion in the final detection task, achieving better detection efficiency. EA-LSS [23] leverages the depth and edge information from point cloud data to provide more accurate depth prediction capabilities for image LSS, thereby obtaining more precise BEV representations. The above methods achieve feature fusion by spatially aligning point cloud features and image features, but they are sensitive to bias of sensor calibration and configuration. Cross Model Transformer [15] (CMT) avoids the bias caused by explicit view transformation through coordinate encoding, while improving robustness to multi-sensor configurations.

The decision-level fusion model has the lowest utilization of multi-modal data, with its focus not on designing fusion strategies for different features, but rather on improving model performance by designing algorithms to filter predicted bounding boxes from visual and point cloud methods.

Currently, multi-modal fusion methods primarily focus on designing effective fusion strategies that combine features from two modalities, while neglecting the fine-grained information that could enhance the model’s expressive capabilities. In complex feature fusion processes and deep network learning, such details are often severely lost, leading to a decline in detection accuracy. Therefore, this paper proposes the IFE-CMT model, which introduces an Instance feature Enhancement module (IE-Module) to provide object-level feature representations for network learning, thereby enhancing fine-grained features.

## 3. Method

**Figure 1:**
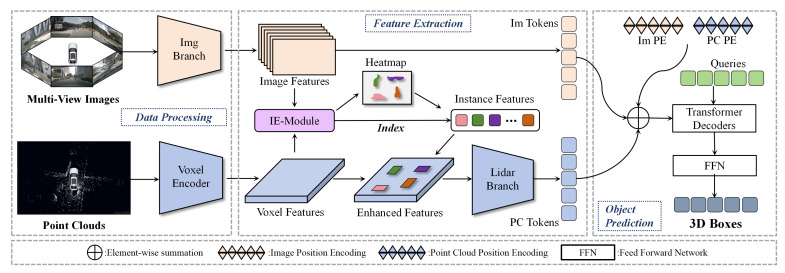
The overall structure of IFE-CMT is shown in the figure. Multi-view images extract image features through image branches and point clouds extract point cloud features through sparse voxel encoders. These point cloud features are enhanced by our IE-module and further processed by our improved point cloud branch. Finally, they are sent to the detection head together with image features for detection.

In Figure 1, this paper presents the overall architecture of the proposed IFE-CMT model. We designed an Instance feature Enhancement module (IE-Module) and optimized the point cloud branch network. This model can learn more fine-grained feature representations while retaining multi-scale information and a larger receptive field.

The model implementation can be divided into four stages: Firstly, multi-view image features are extracted using a pre-trained image backbone network, which provides prior knowledge for the point cloud branch learning and accelerates training. Point clouds are first efficiently converted into point cloud features using a sparse voxel encoder. Secondly, the obtained image features and point cloud features are jointly fed into IE-Module for processing. This step aims to obtain object features and combine them with the point cloud features to enhance the model’s fine-grained information representation. Thirdly, our improved point cloud branch network is used to extract features from point features that has been enhanced with instance features. The improved point cloud network effectively expands the network’s receptive field while enhancing the texture information of object instances through the fusion of shallow features, thereby significantly improving feature expression capabilities. Finally, the image features and enhanced point cloud features are used to perform 3D bounding box regression through a PETR-like detection head.

### 3.1. Instance Feature Enhancement Module (IE-Module)

Most existing multi-modal object detection algorithms primarily leverage scene information, often neglecting the finer-grained features inherent in object information. In fact, this information can effectively help model improve ability for small and easily confused objects’ detection, such as pedestrians, motorcycles, bicycles, etc., without adding too much computational burden to the network.

**Figure 2:**
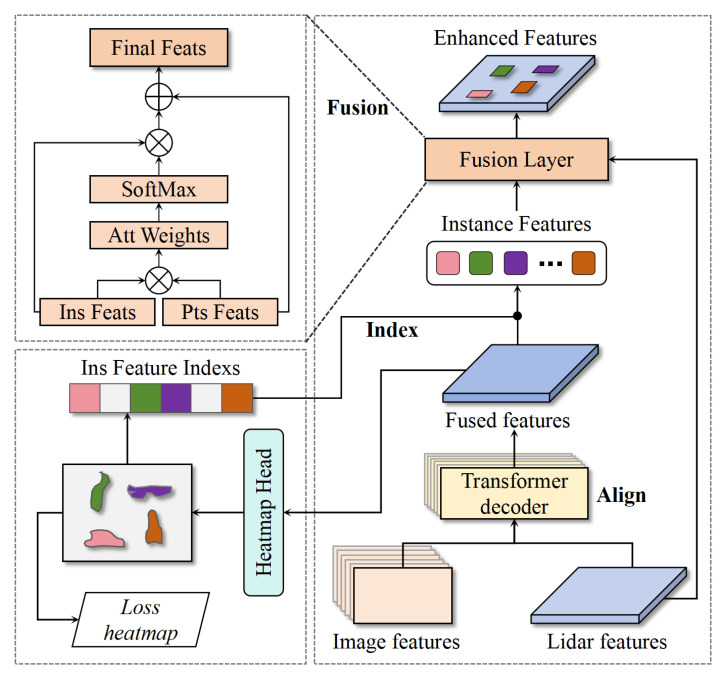
The overall structure of the IE-Module. It mainly consists of three steps: align, index, and fusion. The align part uses self-learning attention to fuse multi-modal features, the index part uses instance heatmap indexs to obtain instance features, and the fusion part uses the attention mechanism to combine instance features and point cloud features. Here, ‘⊗’ denotes element-wise multiplication, and ‘⊕’ denotes element-wise summation.

To address this issue, this paper proposes the IE-Module, which can extract multi-modal object features with rich semantic information to enhance the scene features of point cloud data. As shown in Figure 2, the module first receives image features extracted by a pre-trained network and point cloud voxel features encoded by sparse voxel encoding, then aligns the multi-modal features through a simple self-learning decoder to obtain multi-modal fusion features. The fused features incorporate both the precise spatial information of point cloud features and the rich semantic features of image features, providing a more comprehensive instance feature representation for subsequent tasks.

We designed a heatmap generator consisting of a series of 2D convolution layers. The fused features are processed by this heatmap generator to generate corresponding heatmaps containing location information for various categories. After clip-sigmoid normalization, the heatmap loss is calculated with the ground truth heatmap. We introduced a Gaussian focus loss function [45] to optimize it, and the mathematical expression is as follows:

(1) $$Loss_{Pos}=w_{1}\cdot \sum_{(x,y)\in Pos}{\left(1-pred\right)}^{\alpha }\cdot \left(-\log\left(pred\right)\right)$$

(2) $$Loss_{Neg}=w_{2}\cdot \sum_{(x,y)\in Neg}{\left(pred\right)}^{\alpha }\cdot \left(-\log\left(1-pred\right)\right)$$

(3) $$Loss_{heatmap}=\frac{1}{N}\left(Loss_{Pos}+Loss_{Neg}\right)$$

In these formulas, Pred represents the normalized prediction heatmap. The parameter α denotes the weight used to adjust the loss of easy and hard samples when calculating the loss for positive and negative samples. $w_{1}$ denotes the weighted factor of the positive sample region, while $w_{2}$ indicates the weighted factor of the negative sample region. These weights are derived from Gaussian distribution matrices and are employed to enhance the model’s sensitivity to critical positions while mitigating the influence of negative samples. Additionally, *N* signifies the number of positive samples in the ground truth heatmap, which is utilized for loss normalization.

The computation of Gaussian Focal Loss can be divided into three steps. First, Equation (1) traverses the locations of all positive samples, that is, the object regions of the ground-truth heatmap, and calculates the standard cross-entropy loss for each location as the basic loss for positive samples. The focal modulation factor ${\left(1-pred\right)}^{\alpha }$ is the core of the focal loss. For easily predictable objects, the larger the pred value, the smaller the modulation factor, thereby suppressing the contribution of easy positive samples to the loss value and enabling the model to focus more on hard-to-classify samples. $w_{1}$ is a weight map obtained based on a two-dimensional Gaussian distribution, which is used to increase the loss weight for the key locations of the objects and improve localization accuracy. Then, Equation (2) traverses the locations of all negative samples and similarly calculates the standard cross-entropy loss as the basic loss, applying the focal modulation factor ${\left(pred\right)}^{\alpha }$ to reduce the contribution of easy negative samples to the total loss. $w_{2}$ is designed to be complementary to $w_{1}$, increasing the loss weight for the background adjacent to the objects’ center and reducing the misclassification probability between the background and objects. Finally, the positive and negative sample losses are added together and normalized to eliminate the impact of differences in the number of objects in different scenes and stabilize the training process.

In this paper, heatmap loss is combined with classification loss and bbox loss according to the corresponding loss weights and optimized together during training:

(4) $$Loss=w_{cls}\cdot Loss_{cls}+w_{bbox}\cdot Loss_{bbox}+w_{heatmap}\cdot Loss_{heatmap}$$

Next, the IE-Module performs Non-Maximum Suppression (NMS) on the obtained instance heatmaps to obtain instance location index information. Using this location index encoding, we can extract instance features corresponding to the object coordinates from the fused features.

Finally, we fuse instance features with scene features using a simple self-attention module. Specifically, we first calculate the similarity between instance features and scene features, normalize the results to obtain attention weights. Then multiply the weight information by the instance features to obtain all features in the instance features that are correlated with the scene features, and finally fuse them with the point cloud scene features. The formula can be expressed as follows:

(5) $$Output=F_{ins}\left(\mathrm{sigmoid}\left(F_{ins}\cdot F_{pts}^{T}\right)\right)+F_{pts}$$

In this formula: $F_{ins}$ refers to the instance features extracted by IE-Module, and $F_{pts}$ refers to the overall features of the point cloud. T is the matrix transposition operation. The sigmoid function is used to confine the attention weights within the range of 0 to 1, facilitating feature weighting.

Through the above operations, we ultimately obtained the point cloud feature representation enhanced with instance features, which contains the semantic information of image features, compensating for the lack of semantic information in point cloud features, while providing a more granular feature representation for the overall features.

Furthermore, the proposed IE-Module structurally adheres to lightweight design principles, achieving enhanced feature representation capabilities through a lightweight architecture and low parameter count. This module avoids explicit spatial transformations, which not only reduces memory consumption but also facilitates ease of use by eliminating the need for additional data preprocessing or coordinate alignment operations. It can be directly integrated into existing mono-modal or multi-modal 3D detection frameworks. Specifically, the IE-Module only requires the 2D features output by the image backbone and the point cloud features. Through implicit cross-modal spatial correlation, it can output enhanced point cloud features in real time during inference, thereby improving the detection performance of the model. The Gaussian Focal Loss can be incorporated into the total loss function in a plug-and-play manner, thus requiring no additional modifications. This enables the IE-Module to achieve seamless plug-and-play integration into any object detection framework.

### 3.2. Improved Point Cloud Branch

In current multi-modal object detection models, the point cloud branch network typically adopts the Feature Pyramid Network (FPN) structure used in the image domain. This structure can handle the scale transformation relationship of ’near large, far small’ in image content, providing the network with multi-scale feature representations and effectively improving model accuracy. However, point cloud data directly reflects the scale information of objects in 3D space, and there are no significant scale differences caused by perspective relationships. Therefore, when extracting point cloud features, expanding the receptive field may be a more effective approach.

**Figure 3:**
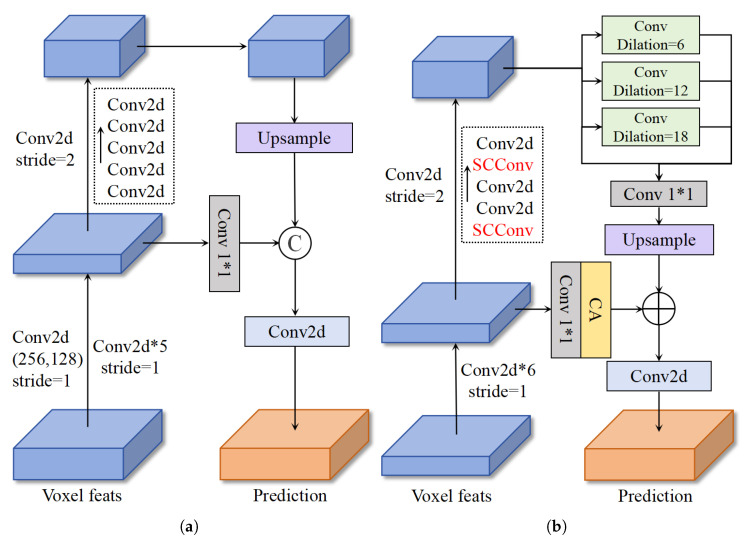
The figure shows the point cloud branch network structure before and after improvement. (**a**) shows the multi-scale structure used in the CMT model. (**b**) shows our improved new architecture that integrates large receptive fields and multi-scale features. In the figure, ‘C’ denotes the concatenation operation of tensors, and ‘CA’ represents the abbreviation for channel attention. The two-stage backbone network is depicted according to the optimal experimental combinations.

The success of PillarNeXt [38] demonstrates that, for 3D point cloud detection tasks, ASPP’s large receptive field is more effective than FPN’s multi-scale features. Although deep-level features possess stronger semantic and global properties, expanding the receptive field via ASPP yields rich features; the absence of object details and texture information can lead to a decrease in the model’s learning speed. In contrast, shallow-level features contain more abundant edge and texture information, which can help the model accelerate convergence in the early stages of training. Therefore, we combined FPN and ASPP into a single neck network structure, as shown in Figure 3. Figure 3a depicts the base FPN structure of the CMT model, while Figure 3b illustrates the new neck network we designed by integrating FPN and ASPP.

As shown in Figure 3b, the point cloud features are passed through a sparse voxel encoder and then fed into the point cloud backbone for processing, yielding two-stage features. The shallow features obtained in the first stage and the deep features obtained in the second stage have different channel numbers and feature map sizes. The advantage of shallow features lies in their retention of more original information and texture details, while the advantage of deep features lies in their stronger semantic representation capabilities and global perspective. Therefore, introducing the ASPP architecture after the second stage enables the acquisition of features with a larger receptive field. Subsequently, the deep features are aligned in feature map size through a 2× upsampling module after ASPP learning. The shallow features are aligned in channels via a 1 × 1 convolution and a simple channel attention module to reduce noise in the shallow features and reduce semantic discontinuity. Finally, the outputs from these two stages are added and fused together. The shallow features supplement the texture information of the ASPP features, which can assist in optimizing the deep features and accelerating training during the early stages of model training, and further enhance the overall feature representation in the later stages.

In addition, the feature redundancy and feature loss in deep neural networks are also major challenges in optimizing large-scale models, often leading to limitations in accuracy. Furthermore, the unique multi-receptive field feature fusion structure of the ASPP network, while covering a broader spatial range, also results in a significant increase in the number of channels, thereby further amplifying the redundancy between spatial and channel features. Our experimental revealed that simply introducing the ASPP network for improvement yields only minimal performance gains. This is because while the receptive field is expanded, it also introduces more redundant features, leading to a decline in feature expression capability.

**Figure 4:**
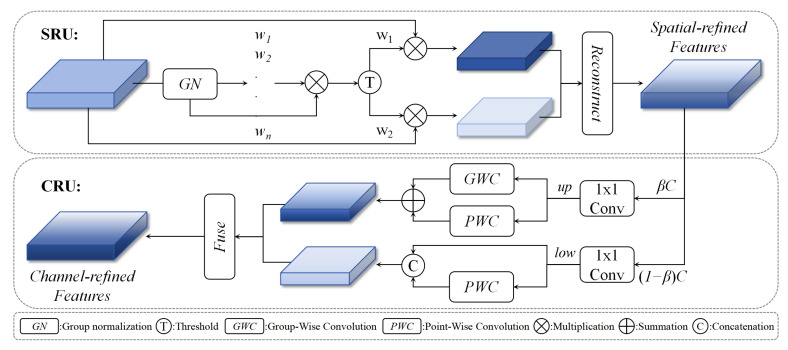
The figure shows the overall structure of SCConv. It includes the Spatial Reconstruction Unit (SRU) and Channel Reconstruction Unit (CRU), which are used to organize features in the spatial and channel dimensions, respectively, to efficiently reduce feature redundancy.

To address this issue, we introduced two layers of Spatial and Channel reconstruction Convolution (SCConv [29]) modules into the point cloud backbone network to reshape spatial and channel features and reduce information redundancy in the network. As shown in Figure 4, the SCConv concept is similar to the spatial and channel attention mechanisms, both of which enhance important feature representations by weighting information. SCConv consists of two units: the Spatial Reconstruction Unit (SRU) and the Channel Reconstruction Unit (CRU), which are used to reshape spatial and channel features, respectively. The SRU separates spatial features through normalized information evaluation and reconstructs spatial features through cross-reconstruction; the CRU separates spatially refined features through the hyperparameter β, where β is within the range of 0 to 1, typically set to 0.5. The channel numbers are divided into βC and (1−β)C, respectively. After 1 × 1 convolution operations, these parts are transformed into up features and low features. The up features are processed as rich features, while the low features are processed as poor features. Then CRU processes rich features using group-wise convolution (GWC) and point-wise convolution (PWC), while processing poor features only by point-wise convolution (PWC). Finally, it integrates the processed features by employing feature normalization weighting to obtain channel-reconstructed features.. By combining SRU and CRU, spatial and channel features can be reconstructed in the intermediate layers of the network, effectively reducing redundant features in deep networks while lowering computational complexity.

In this paper, we replace traditional 2D convolutions with SCConv in the first layer and middle layer of the backbone’s second stage. The SCConv in the first layer is used to reduce the redundant features caused by channel expansion, while the SCConv in the middle layer is used to suppress the further generation of redundant features between spatial and channel dimensions. Through these optimizations, our point cloud branch can simultaneously obtain multi-scale information and a larger receptive field, providing a more comprehensive feature representation for object detection tasks.

## 4. Experiments

### 4.1. Datasets and Metrics

We trained and evaluated our model using the nuScenes dataset [46], a large open-source dataset that provides data from multiple sensors, including six cameras, one LIDAR, five millimeter-wave Radars, an Inertial Measurement Unit (IMU), and GPS. This dataset is widely used in the field of multi-modal fusion algorithms. The dataset comprises 1000 driving scenarios, each containing 20 s of video data, encompassing 10 categories: car, truck, bus, motorcycle, bicycle, trailer, pedestrian, construction vehicle, traffic cones, and barriers. We selected the camera and LIDAR data for our analysis. The camera has a sampling rate of 12 frames per second, with annotations every 0.5 s, while the LIDAR has a sampling rate of 20 frames per second, also with annotations every 0.5 s. We only use these annotated frames.

We evaluate using the official evaluation metrics of the nuScenes dataset: mean Average Precision (mAP), nuScenes Detection Score (NDS). Among them, mAP is obtained by averaging the Average Precision (AP) of each category. Unlike others, the nuScenes dataset employs the distance *d* between center points as the threshold for AP calculation instead of using the Intersection over Union(IoU). As shown in Equation (6), d∈D represents different difficulty settings, with centerpoint distance thresholds set at four levels: 0.5 m, 1 m, 2 m, 4 m. c∈C denotes the calculation of the average AP across different categories. The mAP is obtained by averaging these calculated results.

(6) $$mAP=\frac{1}{\left|C||D\right|}\sum_{c\in C}\sum_{d\in D}{AP}_{c,d}$$

NDS is a weighted fusion of mAP, mean Average Translation Error (mATE), mean Average Scale Error (mASE), mean Average Orientation Error (mAOE), mean Average Velocity Error (mAVE), and mean Average Attribute Error (mAAE). As shown in Equations (7) and (8), the mTP is obtained by averaging the errors of all categories. The NDS metric is derived half from detection accuracy (mAP) and half from detection quality (mTP).

(7) $$mTP=\frac{1}{\left|C\right|}\sum_{c\in C}{TP}_{c}$$

(8) $$NDS=\frac{1}{10}\left[5\cdot mAP+\sum_{mTP\in TP}\left(1-\min(1,mTP)\right)\right]$$

The mAP intuitively reflects the model’s detection capacity, while NDS provides a holistic quantitative assessment of the model’s overall performance.

### 4.2. Implementation Details

We use the ResNet-50 backbone [47] to extract image features, with an input resolution of (800, 320), and employ FPN as the neck network. Our point cloud backbone network utilizes VoxelNet, with point cloud voxelization size of (0.1, 0.1, 0.2), and enhances feature representations using our improved point cloud branch network.

Our model is trained on an RTX4090 GPU with a batch size of 2. The initial learning rate is set to $1\times 10^{-5}$, using the AdamW optimizer with a cyclical learning rate strategy, and the loss weights are set to 2 for classification, 0.25 for bounding-box regression, and 1 for the heatmap. We trained the model for 20 epochs, using GT sample augmentation for the first 15 epochs and without augmentation for the final 5 epochs to further fine-tune the model.

### 4.3. Main Results

To validate our results, we conducted training and testing comparisons on the nuScenes dataset. As shown in Table 2, using the same image resolution, voxel size, and training strategy settings, our model IFE-CMT demonstrates significant performance improvements compared to the baseline (CMT-R50). On the validation set, mAP improved by 2.1% and NDS by 0.8%, while on the test set, mAP improved by 1.9% and NDS by 0.7%.

As shown in Table 3, our method effectively improves the model’s ability to recognize small and easily confused objects, it improves mAP by 6.6%, 3.7%, and 2.1% for the bicycle, motorcycle, and truck categories in the nuScenes dataset, respectively, significantly improving detection accuracy.

Computational complexity remains a critical challenge in deploying 3D object detection models in practical applications. Table 4 summarizes key performance metrics including inference time, memory usage, and detection speed for several mainstream 3D detection models. The baseline CMT network employs a simple and efficient architecture that avoids explicit feature alignment and instead uses positional encoding to implicitly align multi-modal features, thereby reducing model complexity. Furthermore, the incorporation of Flash-attention significantly enhances inference efficiency on GPUs by optimizing parallel computation strategies and reducing non matrix operations, all while maintaining model accuracy. The proposed IFE CMT model improves upon the CMT baseline, and experimental results on the nuScenes dataset demonstrate a notable increase in detection accuracy. Although inference speed is slightly reduced, the model still outperforms most existing multi-modal detection methods and meets the requirements for real-time performance.

**Figure 5:**
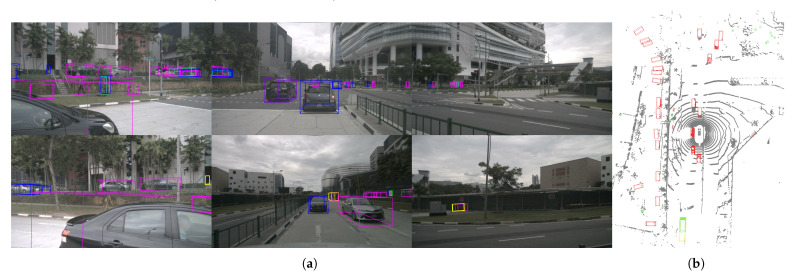
Detection results for omnidirectional cameras and point cloud scenes on the nuScenes validation set. (**a**) shows detection results for the multi-view images. (**b**) shows the detection results for point cloud scene.

Figure 5 shows the detection results of our proposed IFE-CMT in complex road scenes, which can accurately identify objects in the scene and maintain accuracy even for occluded objects and small objects.

**Figure 6:**
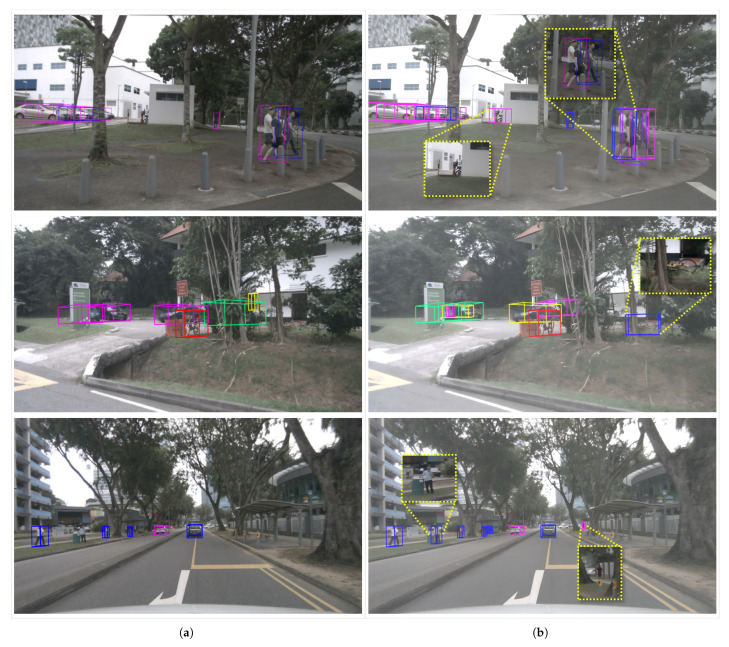
Comparison of visual detection results on the nuScenes validation set. (**a**) shows the detection results of the baseline (CMT-R50), and (**b**) shows the detection results of our method IFE-CMT.

Figure 6 compares the visual detection results of the baseline (CMT-R50) and IFE-CMT on the nuScenes validation set. Figure 6a shows the baseline detection results, while Figure 6b shows the detection results of our IFE-CMT model. As can be seen, in the first set of images, CMT missed the motorcycle obscured by the wall and incorrectly identified three pedestrians gathered together as two. IFE-CMT accurately detected the obscured motorcycle and could accurately separate the gathered crowd. In the second set of images, IFE-CMT also accurately identified the obscured bicycle object. In the third set of images, where there are many small objects, our proposed method accurately identified the pedestrian objects missed by the CMT model. This demonstrates that our proposed improvements can effectively enhance the model’s detection accuracy for small objects, feature-obscured objects, and feature-similar objects.

### 4.4. Ablation Experiment

In this section, we conducted ablation experiments on the nuScenes dataset and compared the results with the baseline (CMT-R50), as shown in Table 5.

When the IE-Module was added, we improve the mAP and nds by 0.8% and a 0.4%. When the point cloud branch were added, we improve mAP and NDS by 1.1% and 0.1%. When both improvements were added together, mAP and NDS have been improved by 2.1% and 0.8%, respectively. This confirms the effectiveness of the improvements we proposed in enhancing the model’s detection capabilities. Next, we will analyze the two improvements separately.

#### 4.4.1. IE-Module

**Figure 7:**
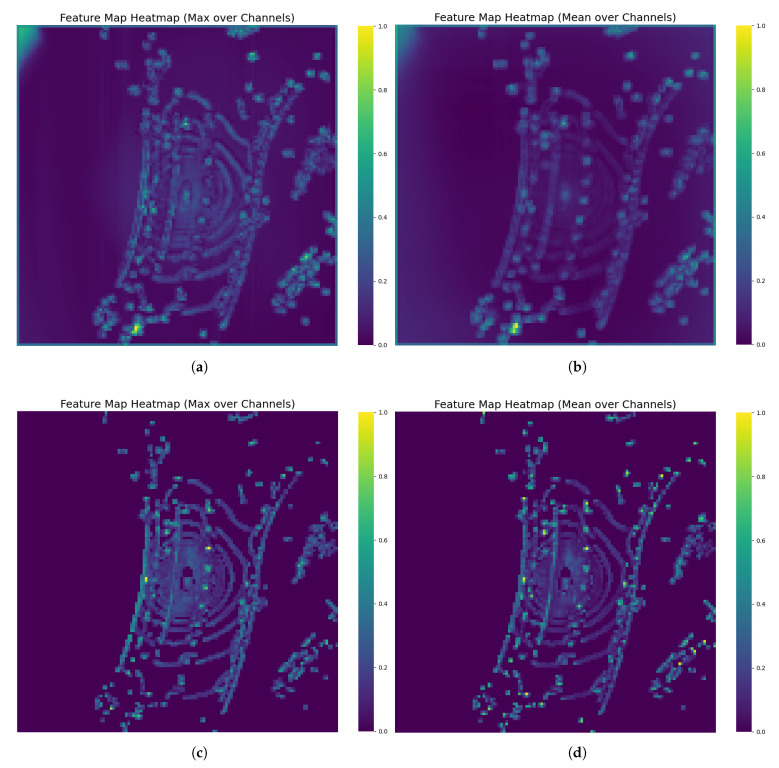
Feature heatmaps before and after adding the IE-Module enhancement. We perform pooling operations on the channel dimension for visualization. (**a**) is the max pooling result when IE-module is not added. (**b**) is the avg pooling result when IE-module is not added. (**c**) is the max pooling result when IE-module is added. (**d**) is the avg pooling result when IE-module is added.

As shown in Figure 7, we perform pooling on the channel dimension to compare feature representations in the network’s intermediate layers. Figure 7a,c show max pooling, while Figure 7b,d show average pooling.

Figure 7a,b show the visualization results before introducing the IE-Module. The heatmap values are relatively dispersed, with insignificant activation differences between the object region and the background, resulting in blurred boundaries and overlap between different instances. Figure 7c,d shows the feature maps after adding the IE-Module at the same network stage. The activation values in the object region are significantly enhanced, the boundaries are clear, and distinct feature isolation zones appear between instances.

The above visualization results qualitatively analyze the effectiveness of IE-module in enhancing instance level feature representation. It can be clearly observed that after the introduction of IE-module, there is a more significant distinction between the object instance and the background. By enhancing the response strength of the object region and suppressing the background noise, the module effectively improves the identifiability and semantics of the feature map. These features significantly promote the extraction and utilization of key information in the subsequent learning process of the model, thus providing a reliable feature basis for improving the detection performance. Therefore, the visual analysis verifies the positive role of IE-module in improving feature representation from a qualitative perspective, and provides an intuitive basis for the performance improvement in the subsequent quantitative analysis results.

In the baseline (CMT-R50) used in this paper, all feature dimensions use 256 channels. The IE-Module we designed includes an attention module, several 2D convolution layers and Transformer decoders. The performance of these intermediate layers is significantly influenced by the number of feature channels. Therefore, to ensure model inference speed, we use 2D convolution in the IE-Module to compress the 256 channel features into 128 channel features for processing. In this section, we conducted comparative experiments to evaluate the performance differences between 128 channel and 256 channel features in terms of detection accuracy and FPS, as shown in Table 6. The experimental results clearly indicate that there is only 0.02% difference in detection accuracy between 128 channel and 256 channel features, but the 256 channel features lag behind by 0.6 FPS in terms of detection speed.

Therefore, based on 128 channel features, the IE-Module we propose improves mAP by 0.8% while only reducing inference speed by 0.2 FPS, offering the advantage of being lightweight.

#### 4.4.2. Improved Point Cloud Branch

The improved point cloud branch network proposed in this paper consists of an improved neck architecture and multiple layers of SCConv. Through extensive experimentation, we have validated the effectiveness of the proposed improvements. As shown in Table 7, the method combining the improved neck architecture with SCConv achieves the best performance improvement. Using ASPP combined with FPN alone results in excessive channel redundancy, leading to a decline in feature expression capability, and the impact on performance is not significant.

Additionally, introducing SCConv with different numbers of layers in the second stage of feature processing also exhibited varying performance. We experimented with replacing the first layer of convolutions, replacing the first and third layers of convolutions, and replacing the first, third, and fifth layers of convolutions. The results indicate that replacing the first and third layers of convolutions achieved the best results. This is because replacing only the first layer of convolutions fails to suppress the generation of redundant features in intermediate layers, while replacing the first, third, and fifth layers introduces feature restructuring operations in the final layer, and the segmentation strategy controlled by CRU hyperparameters may result in the loss of important information. Therefore, replacing the first and third layers achieves the best results.

## 5. Conclusions

This paper aims to address the issues of insufficient utilization of fine-grained features and limited detection accuracy for small objects in existing multi-modal 3D object detection methods, and proposes the IFE-CMT model. This model explicitly enhances the representation of object instances in global features through the Instance feature Enhancement Module (IE-Module), which effectively improves the model’s ability to identify small objects and objects with similar features. Additionally, the module has a simple structure, low computational overhead, and is easy to deploy in any multi-modal/mono-modal object detection model. Compared to the baseline CMT model, the introduction of the IE-Module improves detection accuracy while slightly reducing 0.2 s inference speed. Building on this, we further propose an improved point cloud branch architecture that integrates ASPP and FPN, which expands the network’s receptive field and enhances semantic information while preserving shallow-layer texture features, significantly enhancing the network’s feature representation capabilities. Finally, embedding the SCConv module effectively reduced feature redundancy in the network and improved computational efficiency. Experimental results show that by combining the above improvements, our IFE-CMT model improves mAP and NDS by 2.1% and 0.8% compared to the CMT baseline, confirming the effectiveness of the proposed improvements. The IFE-CMT model also improves mAP for small object categories such as bicycles and motorcycles by 6.6% and 3.7%, respectively, significantly improving detection accuracy for small objects.

Although the effectiveness of the method proposed in this paper has been validated, due to limitations in the current GPU configuration and scale, we are unable to conduct a direct performance comparison with SOTA models. Future work will further explore the following three aspects. Firstly, we will investigate the model’s capacity to integrate with more powerful backbone networks, such as ResNet-101, Swin-Transformer [51], and VoV-Net [52], while also evaluating its performance at higher input resolutions. Secondly, we will pursue a lightweight architectural design that preserves detection accuracy while substantially improving real-time inference performance. Thirdly, we will examine the scalability of the IE-Module when it is incorporated into temporal fusion models.

## Figures and Tables

**Table 1:** Comparison of 3D object detection methods.

Methods		Advantage	Disadvantage	Papers	Limitation
Visual 3D Object Detection	BEV Methods	Achieve more precise object positioning.	The computation is complex and information loss occurs.	[5,6]	Visual methods lack accurate spatial information, resulting in low accuracy.
	Non-BEV Methods	No explicit view transformation, low computational complexity.	Low localization accuracy and weak detection capability.	[7,8]	
Lidar 3D Object Detection	Voxelization Methods	Structured data facilitate processing.	Low resolution leads to loss of fine-grained information.	[9,10]	Lidar point clouds are sparse and semantically weak, hindering scene understanding.
	Transformer Methods	Long-sequence modeling yields high accuracy and rich contextual information.	High computational load and low detection speed.	[11,12]	
Vision-Point Cloud Fusion 3D Object Detection	Data-level Fusion	Full data utilization and complete features.	Relies on visual detection results.	[13,14]	Vision-point-cloud fusion algorithms achieve data complementarity, but existing methods exhibit coarse fusion granularity.
	Feature-level Fusion	Stronger semantics and higher detection accuracy.	Algorithms are complex and computationally intensive.	[15,16]	
	Decision-level Fusion	Algorithm is concise and clear feature.	Low fusion level and poor accuracy.	-	

**Table 2:** Performance comparison on the nuScenes dataset, where C and L represent the use of image data or point cloud data, respectively, and ↑ indicates better performance with increasing values of the metric.

Models	Modality	Resolution	Voxel Size	Backbone	mAP ↑	NDS ↑
Validation dataset						
DETR3D [7]	C	1600 × 900	-	Res101	0.303	0.374
PETR [8]	C	1600 × 900	-	Res101	0.370	0.442
FUTR3D_L [48]	L	-	(0.075, 0.075)	VoxelNet	0.593	0.655
CenterPoint [49]	L	-	(0.075, 0.075)	VoxelNet	0.596	0.668
PointPainting [50]	C & L	-	-	-	0.653	0.685
FUTR3D [48]	C & L	1600 × 900	(0.075, 0.075)	Res101 & VoxelNet	0.645	0.683
Transfusion [16]	C & L	800 × 448	(0.1, 0.1)	Res50 & VoxelNet	0.648	0.691
CMT-R50 [15]	C & L	800 × 320	(0.1, 0.1)	Res50 & VoxelNet	0.655	0.694
Ours	C & L	800 × 320	(0.1, 0.1)	Res50 & VoxelNet	0.676	0.702
Test dataset						
CMT-R50 [15]	C & L	800 × 320	(0.1, 0.1)	Res50 & VoxelNet	0.662	0.696
Ours	C & L	800 × 320	(0.1, 0.1)	Res50 & VoxelNet	0.681	0.703

**Table 3:** Comparison of various types of average accuracy on the nuScenes validation set.

Model	Car	Truck	Construction Vehicle	Bus	Trailer	Pedestrian	Motorcycle	Bicycle	Traffic Cone	Barrier
Baseline	0.864	0.616	0.294	0.729	0.416	0.843	0.727	0.624	0.730	0.706
Ours	0.875	0.637	0.316	0.748	0.424	0.854	0.764	0.690	0.742	0.707
Δ	+1.1%	+2.1%	+2.2%	+1.9%	+0.8%	+1.1%	+3.7%	+6.6%	+1.2%	+0.1%

**Table 4:** Computational-complexity comparison across methods on the nuScenes validation dataset, where C and L represent the use of image data or point cloud data, respectively.

Models	Modality	mAP	NDS	Latency (ms)	Memory (MB)	FPS
FUTR3D_L [48]	L	0.593	0.655	144.2	3515	6.9
CenterPoint [49]	L	0.596	0.668	62.0	2563	16.1
PointPainting [50]	C & L	0.653	0.685	182.0	5290	5.5
FUTR3D [48]	C & L	0.645	0.683	315.7	6477	3.2
Transfusion [16]	C & L	0.648	0.691	153.9	5628	6.5
CMT-R50 [15]	C & L	0.655	0.694	91.8	4298	10.9
Ours	C & L	0.676	0.702	104.2	5196	9.6

**Table 5:** Comparison of ablation experiments with proposed improvements, where ✔ means using the module and ↑ indicates better performance with increasing values of the metric.

IE-Module	Pts Branch	mAP ↑	NDS ↑	FPS ↑
-	-	0.655	0.694	10.9
✔	-	0.663	0.698	10.7
-	✔	0.666	0.695	10.2
✔	✔	0.676	0.702	9.6

**Table 6:** Ablation experiments under different channel number settings of IE-Module, where ↑ indicates better performance with increasing values of the metric.

Method	Channel	mAP ↑	FPS ↑
CMT-R50	-	0.655	10.9
Ours	128	0.663	10.7
	256	0.663	10.1

**Table 7:** Ablation experiments under different point cloud branch settings, where ✔ means using the module, ↑ indicates better performance with increasing values of the metric, and (x,x,x) means SCConv is used to replace Conv2D at layer x.

Neck	SCConv	mAP ↑	NDS ↑
-	-	0.663	0.698
✔	-	0.669	0.698
✔	(1)	0.671	0.699
✔	(1, 3)	0.676	0.702
✔	(1, 3, 5)	0.673	0.701

## Data Availability

Data are contained within the article.
